# Inhibition of DTYMK significantly restrains the growth of HCC and increases sensitivity to oxaliplatin

**DOI:** 10.1038/s41419-021-04375-3

**Published:** 2021-11-18

**Authors:** Fengze Sun, Yuanyuan Liu, Tingting Gong, Qiuzhong Pan, Tong Xiang, Jingjing Zhao, Yan Tang, Hao Chen, Yulong Han, Mengjia Song, Yue Huang, Han Li, Yuanyuan Chen, Chaopin Yang, Jieying Yang, Qijing Wang, Yongqiang Li, Jia He, Desheng Weng, Ruiqing Peng, Jianchuan Xia

**Affiliations:** 1 Department of Biotherapy, State Key Laboratory of Oncology in Southern China, Collaborative Innovation Center for Cancer Medicine, Sun Yat-sen University Cancer Center, Guangzhou, Guangdong China; 2 Department of Ultrasound, The Fifth Affiliated Hospital of Sun Yat-Sen University, Zhuhai, Guangdong China

**Keywords:** Oncogenes, Biomarkers

## Abstract

Most patients with hepatocellular carcinoma (HCC) are in the middle or advanced stage at the time of diagnosis, and the therapeutic effect is limited. Therefore, this study aimed to verify whether deoxythymidylate kinase (DTYMK) increased in HCC and was an effective therapeutic target in HCC. The findings revealed that the DTYMK level significantly increased and correlated with poor prognosis in HCC. However, nothing else is known, except that DTYMK could catalyze the phosphorylation of deoxythymidine monophosphate (dTMP) to form deoxythymidine diphosphate (dTDP). A number of experiments were performed to study the function of DTYMK in vitro and in vivo to resolve this knowledge gap. The knockdown of DTYMK was found to significantly inhibit the growth of HCC and increase the sensitivity to oxaliplatin, which is commonly used in HCC treatment. Moreover, DTYMK was found to competitively combine with miR-378a-3p to maintain the expression of MAPK activated protein kinase 2 (MAPKAPK2) and thus activate the phospho-heat shock protein 27 (phospho-HSP27)/nuclear factor NF-kappaB (NF-κB) axis, which mediated the drug resistance, proliferation of tumor cells, and infiltration of tumor-associated macrophages by inducing the expression of C-C motif chemokine ligand 5 (CCL5). Thus, this study demonstrated a new mechanism and provided a new insight into the role of mRNA in not only encoding proteins to regulate the process of life but also regulating the expression of other genes and tumor microenvironment through the competing endogenous RNA (ceRNA) mechanism.

## Introduction

The 5‐year relative survival rate of liver cancer is only 20%, and the incidence rate is rising annually [1]. Hepatocellular carcinoma (HCC) is the most common type of all liver cancers (comprising 80% of cases). Infection with hepatitis B virus (HBV) and hepatitis C virus, alcoholic liver disease, and most probably nonalcoholic fatty liver disease are major risk factors for HCC. Among them, chronic HBV infection accounts for ~50% of all cases of HCC [2]. Most patients with HCC are in the middle or advanced stage at the time of diagnosis, with a high degree of malignancy, easy recurrence, and poor prognosis, which seriously threatens their health and life [3, 4]. The treatment of HCC often requires multidisciplinary knowledge, including surgery, hepatology, interventional radiology, oncology, and so forth [5, 6]. Orthotopic liver transplantation or surgical resection is considered the most effective treatment; alternative nonsurgical treatments include microwave coagulation therapy, percutaneous acetic acid injection, laser interstitial thermal ablation therapy, radiofrequency ablation, and cryoablation therapy [7, 8]. However, the long-term therapeutic effect of HCC remains unsatisfactory, especially in patients with advanced unresectable disease. Oxaliplatin is one of the most commonly used chemotherapeutic drugs in transcatheter arterial chemoembolization, hepatic arterial infusion, and systemic administration of HCC [9–11], although oxaliplatin resistance is also an important reason for poor therapeutic effect and recurrence of HCC [12, 13]. Hence, finding effective markers and targets of HCC and increasing the sensitivity to drugs are critical to improving the prognosis of patients with HCC.

DNA synthesis is an essential prerequisite for cell replication, especially in tumor cells. Therapeutic agents that target deoxyribonucleoside triphosphate synthesis and metabolism are commonly used in the clinical treatment of several cancer types [14]. The deoxythymidine-5′-monophosphate (dTMP) is synthesized from the methylation of deoxyuridine-5′-monophosphat by thymidylate synthase in the de novo pathway [15]. In the salvage pathway, dTMP is produced from the phosphorylation of thymidine by thymidine kinase. Deoxythymidylate kinase (DTYMK) can catalyze the phosphorylation of dTMP to form dTDP. Besides, it is the first merged step of both salvage and de novo pathways in the production of dTTP, which is an important material for DNA synthesis [16, 17]. Previous studies reported that the knockdown of DTYMK inhibited this pathway, leading to a decrease in the product dTDP and the accumulation of the substrate dTMP [18]. However, nothing else is known, except that DTYMK could catalyze the phosphorylation of dTMP to form dTDP. Few studies have reported on the role of DTYMK, particularly in cancer occurrence and progression. According to the results of The Cancer Genome Atlas (TCGA), the expression of DTYMK increased and associated with a poor prognosis in several cancers. Thus, this study aimed to verify whether DTYMK expression increased in HCC and was an effective therapeutic target in HCC.

## Results

### Increased expression of DTYMK in HCC

**Fig. 1:**
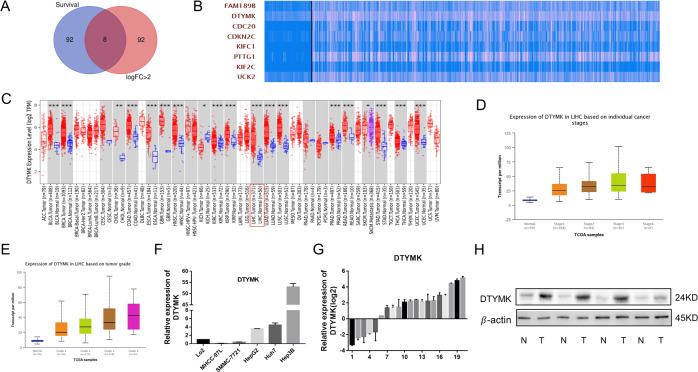
Increased expression of DTYMK in HCC. **A** Gene analysis of patients with HCC from TCGA. **B** Eight genes were upregulated and had the most significant survival differences (**C**) DTYMK expression increased in multiple cancer types, especially in HCC. **D** Expression of DTYMK was related to cancer stages. **E** Expression of DTYMK was related to cancer grades. **F** Increased RNA level of DTYMK in HepG2, Huh7, and Hep3B HCC cell lines. **G** Increased RNA level of DTYMK in most patients with HCC. **H** Increased protein level of DTYMK in most patients with HCC.

After analyzing HCC data from the TCGA, the top 100 mRNAs were upregulated most significantly (fold change > 2), and the top 100 genes with the most significant survival differences were intersected to obtain the critical genes in HCC genesis and development (Fig. 1A). Eight genes fitted the inclusion criteria: FAM189B, DTYMK, CDC20, CDKN2C, KIFC1, PTTG1, KIF2C, and UCK2 (Fig. 1B). Among these genes, this study focused on DTYMK. No in-depth studies on DTYMK have been reported to date. DTYMK was found to be highly expressed in Pan-Cancer Atlas, especially in HCC (Fig. 1C). Besides, the expression of DTYMK was related to tumor stages and grades in HCC according to the results of TCGA (Figs. 1D and E). Whether DTYMK expression increased in HCC was verified by examining 20 HCC tissues and paired adjacent nontumor tissues and HCC cell lines using quantitative polymerase chain reaction (qPCR) and Western blot assays. DTYMK was found to increase in HepG2, Hun7, and Hep3B HCC cell lines compared with LO2 hepatic epithelial cells and in most HCC tissues (Fig. 1F–H).

### Decreased expression of DTYMK inhibited HCC growth and increased sensitivity to oxaliplatin

**Fig. 2:**
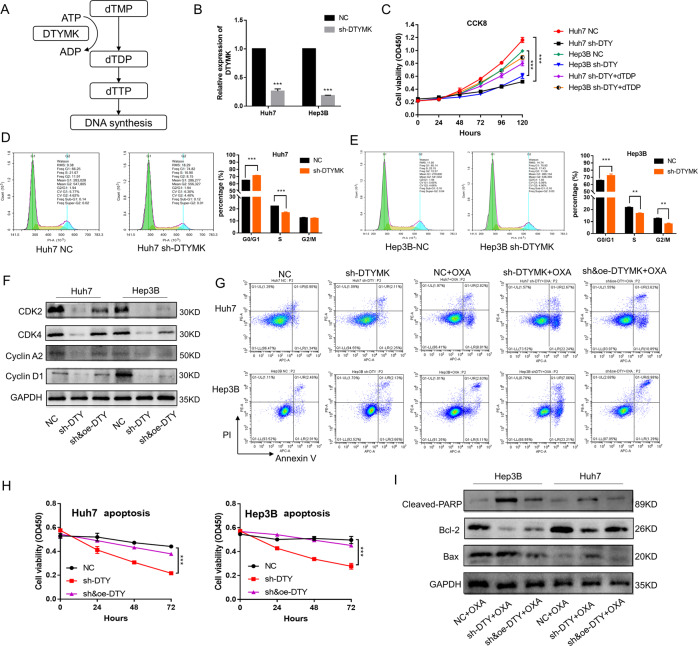
Decreased expression of DTYMK inhibited proliferation and increased sensitivity to oxaliplatin in HCC cells. **A** DTYMK catalyzed the phosphorylation of dTMP to form dTDP. **B** and **C** Decreased DTYMK significantly inhibited the proliferation of Huh7 and Hep3B cells, while the supplement of dTDP partly rescued the proliferation ability. **D** and **E** Decreased expression of DTYMK could lead to cell cycle arrest and reduce the proportion of cells entering the G1 phase. **F** Decreased expression of CDK2, CDK4, Cyclin A2, and Cyclin D1 after DTYMK knockdown. **G** and **H** Increased sensitivity to oxaliplatin after DTYMK knockdown. **I** Reduced Bcl-2 and increased cleaved-PARP and Bax in the sh-DTYMK group than in the other groups. DTY, DTYMK; OXA, Oxaliplatin. ^*^p < 0.05; ^**^p < 0.01; ^***^p < 0.001.

Figure 2A shows how DTYMK was involved in DNA synthesis. After the knockdown of DTYMK, the proliferation of Hep3B and Huh7 HCC cells was significantly inhibited (Fig. 2B and C), while the growth of the tumor cells was partially restored after the addition of dTDP, which was the product of DTYMK. The cell cycle experiment showed that the G0/G1 phase was prolonged and the S phase was shortened after DTYMK knockdown in Huh7 and Hep3B cell lines (Fig. 2D and E). In addition, the levels of cell cycle proteins CDK2, CDK4, Cyclin A2, and Cyclin D1, which were involved in the G0/G1 and S phases, were significantly reduced after DTYMK knockdown (Fig. 2F). Besides, Huh7 and Hep3B cells became more sensitive to oxaliplatin, which was a commonly used chemotherapeutic drug for HCC, after DTYMK knockdown (Fig. 2G and H). The cleavage of Poly ADP-Ribose Polymerase (PARP) during apoptosis has been reported to facilitate cellular disassembly and ensure the completion and irreversibility of the process [19]. Bcl-2/Bax is widely reported as a rheostat that regulates cell death [20]. In this study, cleaved-PARP and Bax increased and Bcl-2 decreased in the sh-DTYMK group than in the other groups (Fig. 2I). The sensitivity to sorafenib also increased after DTYMK knockdown (Supplementary Fig. 1).

### DTYMK acted as ceRNA to affect the function of MAPKAPK2

**Fig. 3:**
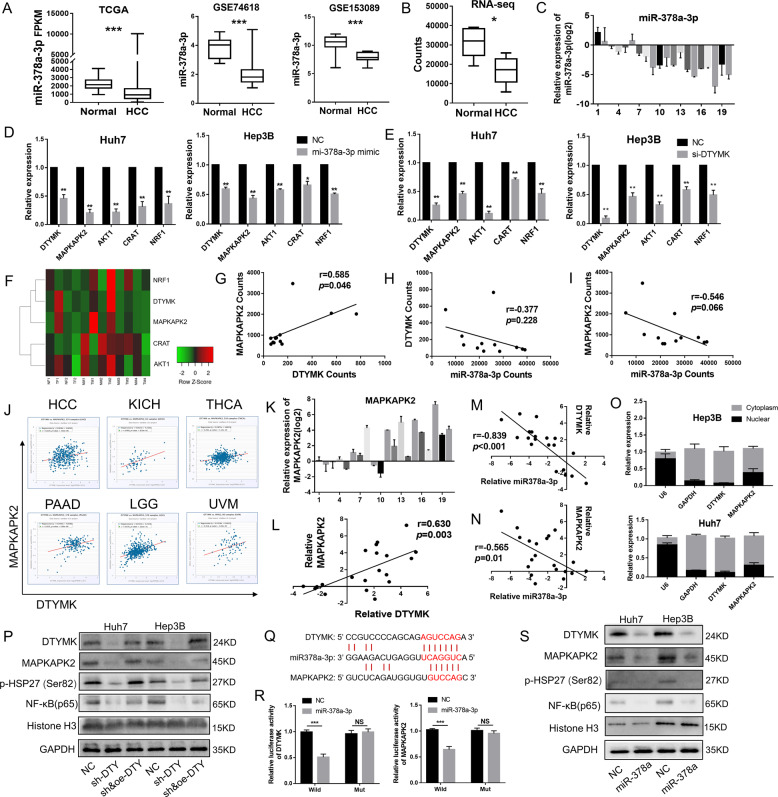
DTYMK acted as ceRNA to affect the function of MAPKAPK2. **A** Decreased expression of miR-378a-3p in HCC from TCGA and GEO. **B** and **C** Decreased expression of miR-378a-3p in HCC from RNA-seq of 6 paired HCC tissues and qPCR of 20 paired HCC tissues. **D** Expression of DTYMK, MAPKAPK2, AKT1, CART, and NRF1 could be inhibited by miR-378a-3p. **E** Decreased expression of MAPKAPK2, AKT1, CART, and NRF1 after DTYMK knockdown. **F** Heatmap (RNA-seq) showed a clear correlation between DTYMK and MAPKAPK2. **G** Positive correlation between DTYMK and MAPKAPK2 (RNA-seq). **H** and **I** Opposite relation between miR-378a-3p, DTYMK, and MAPKAPK2 (RNA-seq). **J** Significant correlation between DTYMK and MAPKAPK2 in multiple cancer types. **K** and **L** Increased expression of MAPKAPK2 in 16 of 20 patients with HCC, which correlated with the expression of DTYMK. **M** An opposite expression trend existed between miR-378a-3p and DTYMK. **N** An opposite expression trend existed between miR-378a-3p and MAPKAPK2. **O** Subcellular locations experiments showing that DTYMK and MAPKAPK2 were enriched in the cytoplasm. **P** Decreased expression of MAPKAPK2 and p-hsp27, and decreased nuclear translocation of NF-κB (p65) after DTYMK knockdown. **Q** Potential binding sites between DTYMK, MAPKAPK2, and miR-378a-3p. **R** MiR-378a-3p bound and significantly inhibited the expression of DTYMK and MAPKAPK2. **S** MiR-378a-3p inhibited the expression of DTYMK, thus inhibiting MAPKAPK2/p-hsp27/NF-κB (p65). ^*^p < 0.05; ^**^p < 0.01; ^***^p < 0.001.

To find out how DTYMK affected the growth of HCC and sensitivity to oxaliplatin, data from TCGA were analyzed using starBASE v2.0 [21]. The results revealed that DTYMK could act as an endogenous competitive RNA (ceRNA) to affect the function of several genes, including MAPKAPK2, AKT1, CART, and NRF1, by binding to microRNA-378a-3p (miR-378a-3p). It was reported that miR-378a-3p could play important roles in cancer development [22]. The results of TCGA, GSE74618, and GSE153089 showed that miR-378a-3p significantly decreased in HCC than in normal tissues[23, 24] (Fig. 3A). Similarly, RNA sequencing (RNA-seq) of six paired HCC and normal liver tissues and qPCR results of 20 paired HCC and normal liver tissues from Sun Yat-sen University Cancer Center showed that miR-378a-3p significantly decreased in HCC (Fig. 3B and C). MAPKAPK2, AKT1, CART, and NRF1 could be inhibited by miR-378a-3p, and their expression decreased after DTYMK knockdown (Fig. 3D and E). According to the clustering analysis of RNA-seq, MAPKAPK2 showed quite similar expression trends with DTYMK (*r* = 0.585, *p* = 0.046) (Fig. 3F and G). Besides, miR-378a-3p with an opposite expression trend of DTYMK (*r* = −0.377, *p* = 0.228) and MAPKAPK2 (*r* = −0.546, *p* = 0.066), although the difference was not significant due to the insufficient number of samples (Fig. 3H and I). Highly relevant expression of DTYMK and MAPKAPK2 was also found in HCC (*r* = 0.229, *p* < 0.001), kidney chromophobe (KICH, *r* = 0.468, *p* < 0.001), thyroid carcinoma (THCA, *r* = 0.281, *p* < 0.001), pancreatic adenocarcinoma (*r* = 0.333, *p* < 0.001), brain lower-grade glioma (*r* = 0.476, *p* < 0.001), and uveal melanoma (*r* = 0.318, *p* = 0.004) (Fig. 3J). MAPKAPK2 expression increased in 15 of 20 HCC tissues (Fig. 3K), which was highly similar to the DTYMK expression (*r* = 0.630, *p* < 0.01) (Fig. 3L). Similar to the RNA-seq, the results of qPCR from the 20 clinical samples showed miR-378a-3p had an opposite expression trend of DTYMK (*r* = −0.839, *p* < 0.001) and MAPKAPK2 (*r* = −0.565, *p* = 0.01) (Fig. 3M and N). In addition, the RNA of DTYMK and MAPKAPK2 both located mainly in the cytoplasm, which was considered as a necessary condition for working through ceRNA (Fig. 3O). MAPKAPK2 could be significantly inhibited after DTYMK knockdown, and thereby inhibit the phosphorylation of heat shock protein 27 (HSP27), which could promote nuclear factor NF-kappaB (NF-κB) in the nucleus (Fig. 3P). Potential binding sites between DTYMK, MAPKAPK2, and miR-378a-3p are shown in Fig. 3Q. Fluorescence intensity was evaluated using dual-luciferase reporter assay after co-transfection with miR-378a-3p mimics to investigate whether miR-378a-3p could inhibit DTYMK and MAPKAPK2 (Fig. 3R). The Western blot analysis showed that miR-378a-3p could significantly inhibit DTYMK/MAPKAPK2/p-HSP27 and prevent NF-κB in the nucleus (Fig. 3S).

### Tumor formation rate and growth speed were significantly inhibited after DTYMK knockdown in vivo

**Fig. 4:**
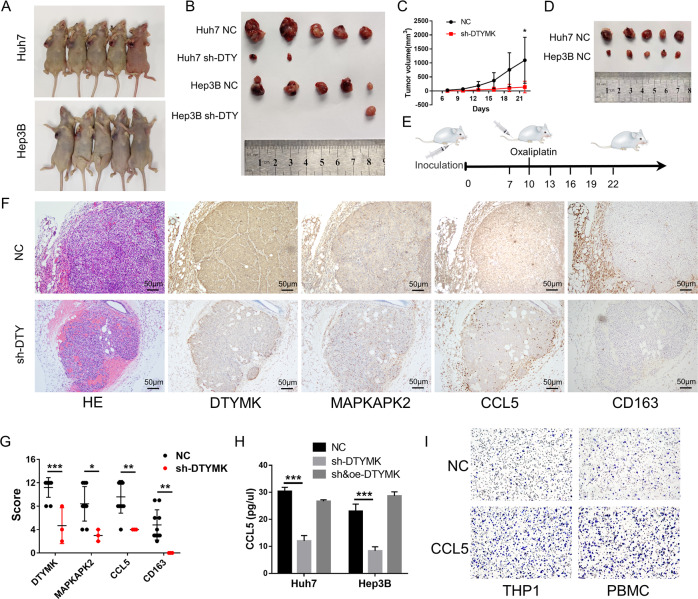
Tumor formation rate and growth speed were significantly inhibited after DTYMK knockdown in vivo. **A** and **B** A stronger inhibitory effect on tumor formation rate was exhibited in transplanted carcinoma in nude mice (*n* = 5 per group). **C** Tumor growth speed was significantly inhibited after DTYMK knockdown (*p* = 0.039). **D** Tumor sizes in the negative control group decreased partially after oxaliplatin treatment, while no tumors were formatted in the sh-DTYMK group (*n* = 5 per group). **E** Schematic diagram of tumor xenotransplantation model. **F** The results of immunohistochemistry showed that the expression of DTYMK and MAPKAPK2 reduced in the sh-DTYMK group compared with the negative control group. Moreover, higher CCL5 expression and more CD163^+^ TAMs were found in the negative control group. Scale bar = 50 μm. **G** Statistical analysis of the mice IHC results. **H** ELISA showed an inhibited level of CCL5 after DTYMK knockdown. **I** CCL5 could stimulate the chemotactic migration of monocytes. NC negative control, DTY DTYMK. ^*^p < 0.05; ^**^p < 0.01; ^***^p < 0.001.

After DTYMK knockdown, a stronger inhibitory effect on tumor formation rate (tumors formatted in only 3 in 10 mice in the sh-DTYMK group) and tumor growth speed (*p* = 0.039) was observed in transplanted carcinoma in nude mice (Fig. 4A–C). The tumor sizes in the negative control group decreased partially after oxaliplatin injection, while no tumors were formatted in the sh-DTYMK group (Fig. 4D). A schematic diagram of the tumor xenotransplantation model is shown in Fig. 4E. The results of immunohistochemistry showed that the expression of DTYMK and MAPKAPK2 reduced in the sh-DTYMK group compared with the negative control group (Fig. 4F). Besides, more CD163^+^ M2 tumor-associated macrophages (TAMs) were found to infiltrate into the tumor and adjacent stroma. This might be due to the higher expression of C-C motif chemokine ligand 5 (CCL5), which was a powerful chemokine to recruit monocytes and could be regulated by NF-kB, in the negative control group (Fig. 4F). The statistical analyze of the IHC images is showen in Fig. 4G. The enzyme-linked immunosorbent assay (ELISA) showed that the level of CCL5 in cell supernatant decreased after DTYMK-knockdown (Fig. 4H). The migration assay showed that CCL5 could stimulate the migration of human monocyte cell line THP1 and CD14^+^ peripheral blood monocytes (PBMC) (Fig. 4I).

### Increased expression of DTYMK was associated with a poor prognosis

**Fig. 5:**
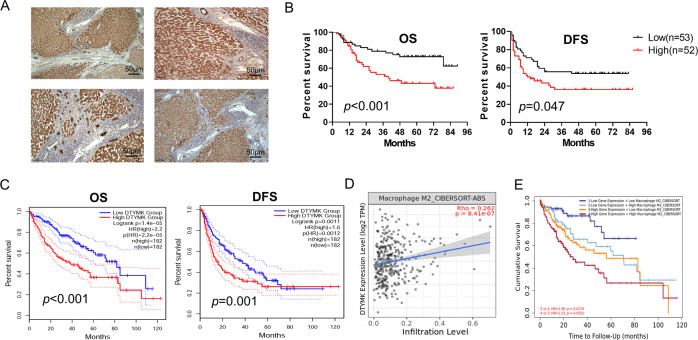
DTYMK expression was significantly upregulated and associated with a poor prognosis. **A** Immunohistochemistry tests showing that DTYMK expression was significantly upregulated in HCC tissues. Scale bar = 50 μm. **B** Increased expression of DTYMK was associated with poor OS and DFS among 105 patients with HCC from the Sun Yat-sen University Cancer Center. **C** Higher expression of DTYMK was associated with poor OS and DFS in HCC from TCGA. **D** and **E** DTYMK correlated with the infiltration of M2 macrophages and predicted the prognosis of patients with HCC more accurately. OS overall survival, DFS disease-free survival.

A total of 105 HCC paraffin-embedded tissues obtained from the Sun Yat-sen University Cancer Center were used to perform immunohistochemical assays to detect protein levels of DTYMK in HCC tissues and adjacent nontumor tissues. The results showed that the protein level of DTYMK was significantly upregulated in HCC tissues compared with adjacent nontumor tissues (Fig. 5A). Higher expression of DTYMK was found to correlate with poorer overall survival (OS) (*p* < 0.001) and disease-free survival (DFS) (*p* = 0.047) (Fig. 5B). Similar results were obtained from the TCGA (Fig. 5C). According to the results of the univariate and multivariate Cox regression analyses, the relative risk of DTYMK was always greater than 1 (*p* = 0.009, *p* = 0.019, respectively), which signified that DTYMK was an independent risk factor for HCC (Table 1). The chi-square test result was shown in Supplementary Table 1. In addition, DTYMK was found to correlate with the infiltration of M2-type macrophages in HCC (*r* = 0.262, *p* < 0.001), which could promote the occurrence and development of tumors (Fig. 5D). The expression of DTYMK and the infiltration of M2-type macrophages were combined to predict the prognosis of patients with HCC more accurately, obtained using TIMER 2.0 [25] (Fig. 5E).

**Table 1:** Univariate and multivariate Cox regression analyses.

Variable	Univariate analysis		Multivariate analysis	
	HR (95% CI)	*p* value	HR (95% CI)	*p* value
Gender		0.977		
Male	1.014 (0.399, 2.578)			
Female	Reference			
Age		0.249		
<50	Reference			
≥50	0.701 (0.382, 1.287)			
HBsAg positive		0.645		
Yes	1.305 (0.403, 4.222)			
No	Reference			
Size (major axis)		0.009		0.068
<5 cm	Reference		Reference	
≥5 cm	2.432 (1.193, 4.955)		2.011 (0.950, 4.259)	
Intact capsule		0.560		
Yes	1.199 (0.654, 2.199)			
No	Reference			
AFP > 400		0.607		
Yes	1.172 (0.642, 2.141)			
No	Reference			
DTYMK expression		0.004		0.019
High	2.470 (1.313, 4.647)		2.157 (1.137, 4.093)	
Low	Reference		Reference	
Liver cirrhosis		0.227		
Yes	1.453 (0.788, 2.680)			
No	Reference			
Grade		0.135		
1	Reference			
2	1.200 (0.488, 2.950)			
3	2.250 (0.861, 5.879)			
TNM staging (AJCC 7th)		<0.001		0.072
I–II	Reference		Reference	
III–IV	5.032 (2.715, 9.328)		2.322 (0.927, 5.814)	
Tumor number (>1)		<0.001		0.092
Yes	4.241 (2.250, 7.994)		2.205 (0.880, 5.525)	
No	Reference		Reference	

## Discussion

With the improvement in molecular biological techniques, some risk factors and molecular mechanisms of HCC have been revealed and several target drugs are being used [26–31]. However, the understanding of HCC is still uncomprehensive and the therapeutic effect is still unoptimistic [32, 33]. The data of HCC were analyzed from the TCGA database for an in-depth understanding of the mechanism of HCC. The expression level of eight genes was significantly increased (fold change > 2) in HCC, including FAM189B, DTYMK, CDC20, CDKN2C, KIFC1, PTTG1, KIF2C, and UCK2, and were associated with poor prognosis. Among these genes, DTYMK was the focus of the present study. Its role in liver cancer was unclear. Only few studies reported on the role of DTYMK, particularly in cancer occurrence and progression. The division and proliferation of tumors cannot be separated from DNA replication [34]. DTYMK has been reported to catalyze the phosphorylation of dTMP to form dTDP, which is an important material in DNA synthesis [18]. Besides, the overexpression of DTYMK has been reported to partially reverse the inhibitory effect of B029-2 on HCC cells, which is a novel p300 inhibitor [35]. In this study, the growth of Huh7 and Hep3B HCC cells and the expression of cell cycle proteins CDK2, CDK4, Cyclin A2, and Cyclin D1, which were involved in the G0/G1 and S phases, significantly reduced after DTYMK knockdown. Besides, Huh7 and Hep3B cells became more sensitive to oxaliplatin and sorafenib after DTYMK knockdown. DTYMK expression was found upregulated in 5-fluorouracil-resistant derivatives, suggesting that DTYMK might be related to drug resistance in colorectal cancer cells [36].

The bioinformatics analysis showed that the expression levels of DTYMK and MAPKAPK2 highly correlated in various tumor types, and they shared the same binding site on miR-378a-3p. Then, miR-378a-3p was found to inhibit the expression of both DTYMK and MAPKAPK2. MicroRNAs (miRNAs) are a large family of posttranscriptional regulators of gene expression, with ~21 nucleotides in length and many developmental and cellular processes controlled by miRNAs in eukaryotic organisms [37]. The discovery of miRNAs opened new doors for the development of novel strategies to combat diseases. Previous studies reported that miR-378a-3p was involved in metabolism, mitochondria, and autophagy [38]. Li et al. reported that miR-378a-3p inhibited the expression of insulin-like growth factor 1 receptor in colorectal cancer cells and might play an important role as a tumor suppressor gene in the initial stage of carcinogenesis of colorectal cancer [39]. MiR-378-3p could also limit the activation of hepatic stellate cells and liver fibrosis by suppressing the expression of Gli3 [40]. No studies have reported that miR-378a-3p could inhibit DTYMK or MAPKAPK2 at present. This study was the first to verify that DTYMK and MAPKAPK2 could combine competitively with miR-378a-3p to maintain the expression of each other, and thus affect phospho-HSP27/NF-κB [41–44]. The HSP27/NF-κB axis is increasingly recognized as a crucial player in many steps of cancer initiation and progression, including immune responses, chemoresistance, and radiation-induced fibrosis [45–48]. The present study indicated that DTYMK mediated oxaliplatin resistance of HCC through the MAPKAPK2/p-HSP27/NF-κB pathway. Survival analysis showed that increased expression of DTYMK was associated with grade, stage, recurrence, and poor prognosis in patients with HCC. Similar to the results in vitro, the tumor formation rate and growth speed were significantly inhibited after DTYMK knockdown in nude mice. Moreover, more CD163^+^ M2-type TAMs were infiltrated in the negative control group than in the DTYMK knockdown group, contributing to the formation and progression of xenograft tumors [49, 50]. Multiple studies reported that CCL5 could recruit monocytes and be regulated by NF-κB; it played essential roles in liver disease progression, especially HCC development in humans and mice [51–54]. DTYMK promoted the expression of CCL5 by affecting NF-κB, which led to increased infiltration of CD163^+^ M2-type TAMs in the tumor microenvironment.

In summary, DTYMK expression was upregulated and was involved in the development of HCC. Increased expression of DTYMK significantly correlated with the poor prognosis in patients with HCC. In addition, DTYMK could competitively combine with miR-378a-3p to maintain the expression of MAPKAPK2 and thus activate the phospho-HSP27/NF-κB axis, which mediated drug resistance, proliferation of tumor cells, and infiltration of CD163^+^ M2-type TAMs by inducing the expression of CCL5. In previous studies, circRNAs and lncRNAs were thought to play a role through the ceRNA mechanism. The present study demonstrated a new mechanism and provided a new insight into the role of mRNA in not only encoding proteins but also participating in the ceRNA mechanism. DTYMK may be a potential biomarker and therapeutic target against HCC.

## Materials and methods

### Patients and specimens

A total of 20 pairs of HCC tissue samples and paired adjacent nontumor tissue samples were collected from the Sun Yat-sen University Cancer Center from 2018 to 2019. A total of 105 paraffin-embedded tissue sections, collected from the Sun Yat-sen University Cancer Center, were used for the immunohistochemical experiment. PBMC were collected from healthy volunteers. All experiments complied with the principles of the Declaration of Helsinki and were approved by the Research Ethics Committee of the Sun Yat-sen University Cancer Center. Informed consent was obtained from all patients to use their tissues for research purposes.

### Cell culture

Hep3B HCC cell line were obtained from American Type Culture Collection (ATCC, Manassas, VA, USA); Huh7 HCC cell line was obtained from the RIKEN cell bank (Ibaraki, Japan). THP1 was provided by Tong Xiang (Sun Yat-sen University Cancer Center). THP1 and CD14^+^ PBMC were cultured in the Roswell Park Memorial Institute-1640 medium (Gibco, NY, USA). Huh7 and Hep3B cells were cultured in the Dulbecco’s modified Eagle’s medium (DMEM) (Gibco, NY, USA) supplemented with 10% fetal bovine serum (FBS) (Gibco, NY, USA) at 37 °C in a humid atmosphere containing 5% CO_2_.

### Transfection

Lipofectamine 3000 (Invitrogen; Thermo Fisher Scientific, Inc.) was used to perform transfections following the manufacturer’s protocol. A total of 7.5 μL of Lipofectamine 3000 and final concentrations of 50 nM of siRNA for DTYMMK (5′-CGAUGUUUAACUCGGUCAACC-3′; 5′-UUGACCGAGUUAAACAUCGUU-3′) were used for each transfection in a six-well plate with 2 mL of the culture medium. Knocked-down DTYMK (target sequence: GTTTCCACCAGCTCATGAA) and negative control lentiviruses were obtained from Shanghai OBIO Technology (Shanghai, China). Huh7 and Hep3B cells were transfected with lentiviruses at a multiplicity of infection of 10. After 2 weeks of 2 µg/mL puromycin screening, Huh7 and Hep3B cells were used for subsequent experiments. Overexpressed DTYMK and negative control plasmid with a resistance of neomycin were obtained from GeneCopoeia (Guangzhou, China).

### RNA isolation and real-time quantitative reverse transcriptase–polymerase chain reaction

Total RNA was isolated using a TRIzol reagent (Invitrogen; Thermo Fisher Scientific, Inc.) following the manufacturer’s protocol. The Fast All-in-One reverse transcription (RT) Kit (cat. no. ES-RT001; Shanghai Yishan Biotechnology, Co., Ltd) was used to perform RT following the manufacturer’s protocol. The relative expression level of mRNAs was normalized to glyceraldehyde-3-phosphate dehydrogenase (GAPDH) and calculated using the 2^−ΔΔCt^ method. The primers used were as follows: DTYMK, forward: 5′-GTCCTGTTCCTCCAGTTAC-3′ and reverse: 5′-AGCATCCACCATCTTCCA-3′; MAPKAPK2, forward: 5′-CGCAGTTCCACGTCAAGTC-3′ and reverse: 5′-GGGCGAATTTCTCCTGGGTC-3′; and GAPDH, forward: 5′-AGAAGGCTGGGGCTCATTTG-3′ and reverse: 5′-AGGGGCCATCCACAGTCTTC-3′.

### RNA and protein isolation from the nucleus and cytoplasm

First, up to 10^7^ fresh cultured cells were collected, washed once with phosphate-buffered saline (PBS), and placed on ice. A PARIS Kit (AM1921, Thermo Fisher Scientific, Inc.) was used to aspirate RNA and protein from the nucleus and cytoplasm. Then, 300 μL of cell disruption buffer was added to half of the cells for total RNA. The other half of cells were resuspended in 300 μL of ice-cold cell fractionation buffer and incubated on ice for 10 min for nuclear and cytoplasm RNA or protein. Then, the samples were centrifuged for 5 min at 4 °C and 500 *g*, and the cytoplasmic fraction was carefully aspirated away from the nuclear pellet. The cytoplasmic lysate was cytoplasmic protein. The nuclear pellet was washed with ice-cold cell fractionation buffer. A cell disruption buffer was used to lyse the nuclear pellet. The lysate was mixed with an equal volume of 2× lysis/binding solution, and a “sample volume” of 100% ethanol was added to the mixture. The sample mixture was drawn through a filter cartridge and washed once with 700 μL of wash solution 1 and twice with 500 μL of wash solution 2. In the end, RNA was eluted with 40 μL of 95 °C elution solution.

### Western blot analysis

The Western blot analysis was performed as described in a previous study [55] using anti-DTYMK antibody (15360-1-AP, Proteintech, IL, USA), anti-MAPKAPK2 antibody (13949-1-AP, Proteintech, IL, USA), anti-GAPDH antibody (60004-1-Ig, Proteintech, IL, USA), anti-phospho-HSP27 (Ser82) antibody (9709 T, Cell Signaling Technology, MA, USA), anti-NF-κB p65 antibody (8242 s, Cell Signaling Technology), and anti-Histone H3 antibody (Cell Signaling Technology).

### Cell proliferation assay

The cell proliferation activity was measured using a Cell Counting Kit-8 (CCK-8, Dojindo Chemical Laboratory, Kumamoto, Japan). Approximately 2 × 10^3^ cells in a 100 μL of medium were seeded into 96-well plates after transfection. After incubation at 37 °C for 2 h, the absorbance at a wavelength of 450 nm was measured following the addition of 10 µL of the CCK-8 solution.

### Dual-luciferase reporter assay

The HEK293T cells were cultured in DMEM supplemented with 10% FBS. Then, luciferase reporter vectors (GeneCopoeia, Guangzhou, China) were transfected into HEK293T cells. Next, 50 nM of miRNA mimics or negative control mimics were transfected into HEK293T cells using Lipofectamine 3000 (Invitrogen; Thermo Fisher Scientific, Inc.). The luciferase activity was detected using the Dual-Luciferase Assay Kit (Promega, WI, USA) after 48 h. The relative luciferase activity was normalized to Renilla luciferase activity. The bases binding to miRNA were replaced by a complementary base for mutation.

### Enzyme-linked immunosorbent assay

A Human CCL5 Quantikine ELISA Kit was purchased from R&D (DRN00B). The ELISA measurement was carried out strictly according to the standard protocol provided by the manufacturer.

### Migration

Migration experiment was carried out using the Falcon permeable support with a 3.0 µm transparent PET membrane. A total of 1 × 10^6^ THP1 or CD14^+^ PBMC (separation by CD14 MicroBeads, Miltenyi) resuspended in 200 μL serum-free 1640 medium were seeded into the upper chamber, and a total of 500 μL of 1640 medium supplemented with CCL5 (10 μg/mL) was added to the lower chamber. After incubation at 37 °C with 5% CO_2_ for 4 h, the cells that passed through the membrane were fixed with 4% formaldehyde for 30 min and washed with PBS two times, and then stained with 0.1% crystal violet for 20 min and washed with water.

### Animal experiments

Five-week-old female BALB/c-nu/nu mice, purchased from Guangdong Medical Laboratory Center (China), were randomly divided into four groups (*n* = 5 in each group). A total of 2 × 10^6^ DTYMK-knockdown Hep3B or Huh7 cells in 0.15 mL of PBS were subcutaneously injected into the right armpit region of the mice, and negative control cells were subcutaneously injected into the other side. The tumor size was measured every 3 days from the 7th day after injection. Oxaliplatin (5 mg/kg) was injected through the tail vein every 3 days from the 10th day after injection. After 28 days of injection, the mice were sacrificed, and tumors were isolated and measured. Animal experiments were approved by the animal ethics committee of the Sun Yat-sen University Cancer Center.

### Statistical analysis

Statistical analyses were performed using SPSS 20.0 (IBM Corp). Graphs were generated using GraphPad Prism 7 (GraphPad Software, Inc). Pan-cancer view and association analysis between DTYMK expression, stages, and grades were performed by UALCAN [56]. Survival analysis of data from TCGA was performed by GEPIA [57]. The unpaired-sample Student *t* test was used to evaluate the differences between the two groups of independent samples. Data were presented as mean ± standard deviation. All the results were repeated more than three times. A *p* value < 0.05 from a two-tailed test was considered to indicate a statistically significant difference. Statistical significance was indicated as ^*^p < 0.05, ^**^p < 0.01, and ^***^p < 0.001.

## Supplementary information

The sensitivity to sorafenib increased after DTYMK knockdown

Supplementary file

Reproducibility checklist

## Data Availability

All data generated during this study and datasets used and/or analyzed during the current study are available from the corresponding author on reasonable request.
