# Supplementary figures and images for: Inhibition of DTYMK significantly restrains the growth of HCC and increases sensitivity to oxaliplatin
Source: Cell Death Dis. 2021 Nov 18;12(12):1093. doi: 10.1038/s41419-021-04375-3 (PMC8602592; doi:10.1038/s41419-021-04375-3)

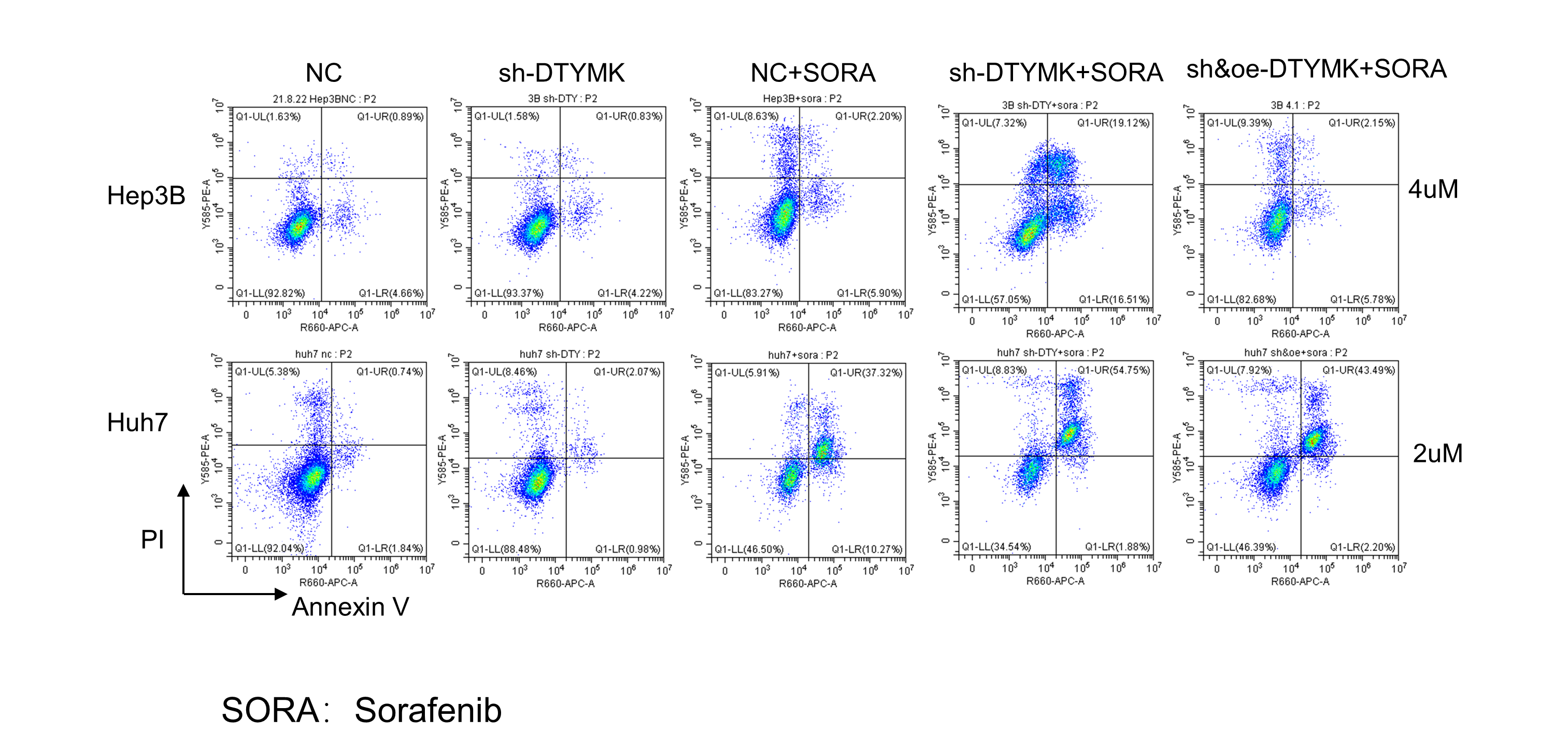

Supplement: Supplementary file 1 — The sensitivity to sorafenib increased after DTYMK knockdown [file 41419_2021_4375_MOESM1_ESM.tif]
