# Supplementary material for: Inhibition of DTYMK significantly restrains the growth of HCC and increases sensitivity to oxaliplatin
Source: Cell Death Dis. 2021 Nov 18;12(12):1093. doi: 10.1038/s41419-021-04375-3 (PMC8602592; doi:10.1038/s41419-021-04375-3)
Supplement: Supplementary file 2 — Supplementary file [file 41419_2021_4375_MOESM2_ESM.docx]

|  | **High** | **Low** |  |
| --- | --- | --- | --- |
| **Variable** | **n=52** | **n=53** | ***p* value** |
| **Gender** |  |  | 0.972 |
| Male | 46 (88.5%) | 47 (88.7%) |  |
| Female | 6 (11.5%) | 6 (11.3%) |  |
| **Age (years)** |  |  | 0.284 |
| <50 | 24 (46.2%) | 30 (56.6%) |  |
| ≥50 | 28 (53.8%) | 23 (43.4%) |  |
| **Recurrence** |  |  | 0.139 |
| Yes | 32 (61.5%) | 25 (47.2%) |  |
| No | 20 (38.5%) | 28 (52.8%) |  |
| **Intact capsule** |  |  | 0.782 |
| Yes | 20 (38.5%) | 19 (35.8%) |  |
| No | 32 (61.5%) | 34 (64.2%) |  |
| **Median survival month** | 40 | - |  |
| **Macrovascular invasion** |  |  | 0.488 |
| Yes | 5 (9.6%) | 3 (5.7%) |  |
| No | 47 (90.4%) | 50 (94.3%) |  |
| **Size (major axis)** |  |  | 0.739 |
| <5cm | 18 (34.6%) | 20 (37.7%) |  |
| ≥5cm | 34 (65.4%) | 33 (62.3%) |  |
| **HBsAg positive** |  |  | 0.093 |
| Yes | 45 (86.5%) | 51 (96.2%) |  |
| No | 7 (13.5%) | 2 (3.8%) |  |
| **AFP>400** |  |  | 0.776 |
| Yes | 24 (46.2%) | 23 (43.4) |  |
| No | 28 (53.8%) | 30 (56.6) |  |
| **Liver cirrhosis** |  |  | 0.926 |
| Yes | 27 (51.9%) | 28 (52.8%) |  |
| No | 25 (48.1%) | 25 (47.2%) |  |
| **Tumor number (>1)** |  |  | 0.435 |
| Yes | 12 (23.1%) | 9 (17.0%) |  |
| No | 40 (76.9%) | 44 (83.0%) |  |
| **Grade** |  |  | 0.210 |
| 1 | 11 (21.2%) | 6 (11.3%) |  |
| 2 | 26 (50.0%) | 35 (66.0%) |  |
| 3 | 15 (28.8%) | 12 (22.7%) |  |
| **TNM staging**  **(AJCC 7th)** |  |  | 0.148 |
| I-II | 37 (71.2%) | 44 (83.0%) |  |
| III-IV | 15 (28.8%) | 9 (17.0%) |  |

Supplementary Table 1. Results of chi-square test.

**Supplementary figure legends**

**Supplementary Figure 1.** The sensitivity to sorafenib increased after DTYMK knockdown.
